# Supplementary material for: Novel Driver Strength Index highlights important cancer genes in TCGA PanCanAtlas patients
Source: PeerJ. 2022 Aug 11;10:e13860. doi: 10.7717/peerj.13860 (PMC9375969; doi:10.7717/peerj.13860)
Supplement: Supplemental Information 2 [file peerj-10-13860-s002.zip › COHORTS/genes plots/cohorts/2021_8_16_14_9_distribution_cohorts_BRCA.pdf]

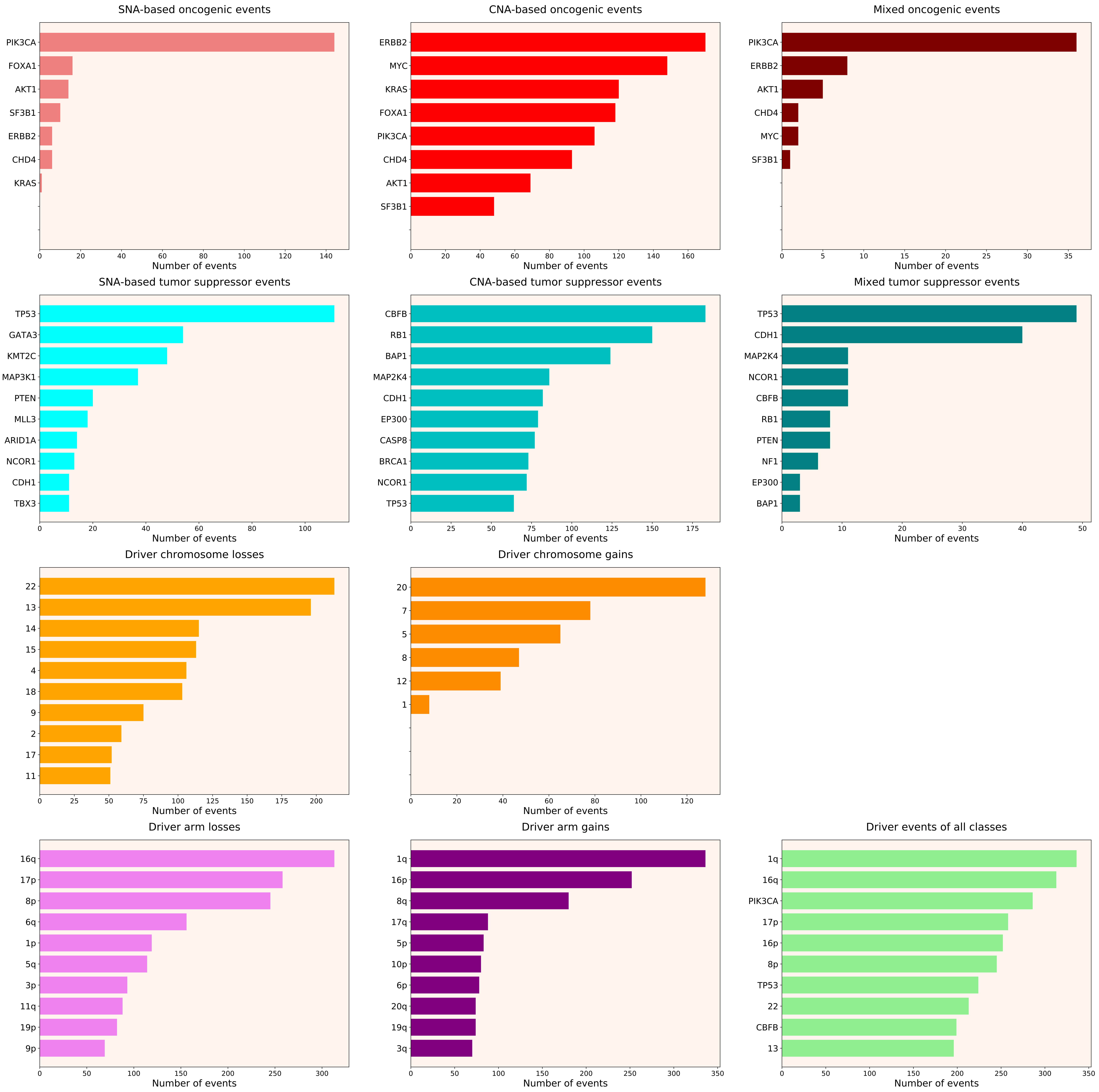

SNA-based oncogenic events
PIK3CA
FOXA1
AKT1
SF3B1
ERBB2
CHD4
KRAS
0 20 40 60 80 100 120 140
Number of events
CNA-based oncogenic events
ERBB2
MYC
KRAS
FOXA1
PIK3CA
CHD4
AKT1
SF3B1
0 20 40 60 80 100 120 140 160
Number of events
Mixed oncogenic events
PIK3CA
ERBB2
AKT1
CHD4
MYC
SF3B1
0 5 10 15 20 25 30 35
Number of events
SNA-based tumor suppressor events
TP53
GATA3
KMT2C
MAP3K1
PTEN
MLL3
ARID1A
NCOR1
CDH1
TBX3
0 20 40 60 80 100
Number of events
CNA-based tumor suppressor events
CBFB
RB1
BAP1
MAP2K4
CDH1
EP300
CASP8
BRCA1
NCOR1
TP53
0 25 50 75 100 125 150 175
Number of events
Mixed tumor suppressor events
TP53
CDH1
MAP2K4
NCOR1
CBFB
RB1
PTEN
NF1
EP300
BAP1
0 10 20 30 40 50
Number of events
Driver chromosome losses
22
13
14
15
4
18
9
2
17
11
0 25 50 75 100 125 150 175 200
Number of events
Driver chromosome gains
20
7
5
8
12
1
0 20 40 60 80 100 120
Number of events
Driver arm losses
16q
17p
8p
6q
1p
5q
3p
11q
19p
9p
0 50 100 150 200 250 300
Number of events
Driver arm gains
1q
16p
8q
17q
5p
10p
6p
20q
19q
3q
0 50 100 150 200 250 300 350
Number of events
Driver events of all classes
1q
16q
PIK3CA
17p
16p
8p
TP53
22
CBFB
13
0 50 100 150 200 250 300 350
Number of events
